# Does Having a Sibling Affect Autistic People's Empathy?

**DOI:** 10.1007/s10803-023-06153-w

**Published:** 2023-11-07

**Authors:** Yonat Rum, Ofer Golan, Carrie Allison, Paula Smith, Simon R. White, Simon Baron-Cohen

**Affiliations:** 1 Autism Research Centre, Department of Psychiatry, University of Cambridge, Cambridge, UK; 2 School of Education, The Hebrew University of Jerusalem, Jerusalem, Israel; 3 Department of Psychology, Bar-Ilan University, Ramat-Gan, Israel; 4 Department of Psychiatry, University of Cambridge, Cambridge, UK

**Keywords:** Siblings, Autism, Empathy

## Abstract

This study examined whether autistic people with siblings score higher on measures of empathy than those without siblings. Cohorts of autistic children (*n* = 939; mean age = 7.35 years (*SD* = 2.15)) and autistic adults (*n* = 736; mean age = 37 years (*SD* = 12.39)) from the Cambridge Autism Research Database (CARD) were each divided into two groups: with or without siblings. Empathy was measured using the children version of the Empathy Quotient (EQ) (parent-report) for children. For adults, the EQ (self-report version) and the Reading the Mind in the Eyes Test (RMET) were used. Contrary to the hypothesis, autistic children without siblings scored higher on EQ than those with siblings (*t*_(283.70)_ = 4.20, p < .001; *d* = 0.50). In adults, there was no difference between autistic adults with and without siblings on both measures, but there was an interaction effect between sex and group on the RMET (*f*_(1732)_ = 4.10, *p* = 0.04): whilst autistic males without siblings on average scored lower than females, autistic males with siblings on average performed similarly to females. Future research should investigate the possible effect of siblings on autistic males' empathy performance in a larger cohort of autistic individuals. Children's empathic abilities may be underestimated by their parents when they have siblings due to a contrast effect.

Sibling relationships are often the most enduring life-long relationships, influencing development (Brody, 2004; Dunn, 2007). Growing up with siblings offers children exposure to social-cognitive growth contexts, such as shared imaginative play, handling conflicts, and practicing reciprocal interaction (Dunn, 2002; Foote & Holmes-Lonergan, 2003; Randell & Peterson, 2009). These contexts are associated with the development of “theory of mind” (ToM) (Hughes & Leekam, 2004; Jenkins & Astington, 1996; Lillard, 1993), that is, the ability to infer and interpret mental states in oneself and others (Wellman, 2002). ToM overlaps with the concept of cognitive empathy. Empathy, broadly defined as the ability to understand and share others’ emotions while maintaining a self-other distinction, underlies successful interpersonal relations (Decety et al., 2016; Uzefovsky & Knafo-Noam, 2016). Researchers point to two components included in this multifaceted concept: cognitive empathy—the intellectual/imaginative apprehension and understanding of others’ emotions, and emotional, or affective empathy—the emotional response to other’s emotion with a similar or an appropriate emotion (Baron-Cohen, 2011; Baron-Cohen & Wheelwright, 2004; Lawrence et al., 2004). Indeed having a sibling is associated with various aspects of social cognitive development in neurotypical children, including empathy (Jambon et al., 2019) and specifically cognitive empathy, or ToM (e.g., McAlister & Peterson, 2007, 2012; Perner et al., 1994).

Recently it was found that the association between having a sibling and ToM in typical individuals, might also extend into adulthood. Lo and Mar (2022) examined a large sample of adults in a cohort collected from the general population (*N* = 1792; *M*_*age*_ = 24.12 years) and found that adults with more older siblings performed better in an age-appropriate task designed to measure ToM, or cognitive empathy (the Reading the Mind in the Eyes Test; RMET; Baron-Cohen et al., 2001). These findings support the theory of a positive effect of having siblings on social-cognitive outcomes in typical individuals.

As siblings also compete over resources such as parental attention and care, another theory would suggest a negative effect of having siblings on social-cognitive development through a process of resource dilution (Downey, 2001; Lawson & Mace, 2009). In families of autistic individuals, this resource dilution hypothesis might be particularly relevant, as parents might have to allocate more resources to support the needs of their autistic child. Considering that challenges in social communication are a primary characteristic of autism, examining the role of siblings and their potential effect on the cognitive-social development and functioning of autistic individuals is important.

Some studies support the hypothesis of the positive effects of siblings on autistic individuals. Rosen et al. (2022) found a positive effect of siblings on growth in adaptive functioning (the ability to meet age-appropriate demands in everyday life) of autistic individuals from childhood to adulthood. Their study included 208 participants (77% reported having at least one sibling) followed over 17 years (from ages 9 to 26) in a longitudinal design. Adaptive functioning was measured using the Vineland Adaptive Behavior Scales (VABS; Sparrow et al.,  1984, 2005). The researchers found that participants with siblings, regardless of birth order position, showed significantly steeper adaptive skill growth trajectories from childhood through adulthood compared to participants without siblings. The authors suggested that siblings may have an important role in improving adaptive functioning trajectories and overall outcomes of autistic individuals.

This finding is consistent with previous literature documenting the positive effect of siblings in areas of social-cognitive functioning and social communication. In a retrospective study, Ben-Itzchak et al. (2016) analyzed records of 112 autistic children (with a mean age of 2.6 years ± 9.2 months; 15 girls and 99 boys) who either had siblings (n = 56, six girls) or did not have siblings (n = 56, seven girls). All participants were evaluated at a tertiary center that provides diagnosis and intervention services and is involved in autism research. The researchers compared the two groups on the Autism Diagnostic Interview-Revised (ADI–R; Rutter et al. 2003), Autism Diagnostic Observation Scales (ADOS; Lord et al. 1999), and VABS (Sparrow et al. 1984). They found that the group with older sibling/s showed lower scores than the group without sibling/s on the ADOS’ social affect sub-domain and the ADI-R communication subdomain scores. These results indicated less severe social-communication difficulties for the group with at least one older sibling than for the group without siblings. This positive sibling effect was replicated in another study (Ben-Itzchak et al., 2019). Autistic participants (n = 150; mean age = 4:0 ± 1:6) were divided into three equal groups (having no siblings, having older siblings, or having younger siblings). The study found that autistic children with older siblings showed fewer challenges in social interaction and better social adaptive skills than only children. These studies support the hypothesis that neurotypical siblings positively impact the social and communicational skills of autistic children. Possible explanations for such findings might be modeling by the neurotypical sibling and the fact that siblings may provide a built-in social companion and communication partner to practice social skills (Rum et al., 2021).

Some studies have explicitly examined the relationships between siblings and the development of ToM in autistic children. Using a battery of four false belief tasks to measure ToM, Matthews et al. (2013) found that autistic children (age 4–12 years old) with at least one older sibling (n = 12) outperformed autistic children with no older siblings (n = 28). In contrast, O’Brien et al. (2011) did not find an association between having siblings and higher ToM scores in their sample of autistic children (n = 60; 90% males; 3–12 years old). This study compared the performance of autistic participants with sibling/s (n = 45) to those without siblings (n = 15) on a standard six-task ToM battery (Wellman & Liu, 2004). Furthermore, their results suggested there was a disadvantage in having an older sibling and a marginally significant advantage in having a younger sibling. However, sample sizes were small in these analyses (n = 15 only-children; n = 13 children with older siblings; n = 22 children with younger siblings; n = 10 children with both older and younger siblings). The wide age ranges in these studies may have hampered attempts to clarify the association between having siblings and performance on ToM tasks in autistic children. Matthews and Goldberg (2018) attempted to address this limitation by examining the effect of siblings on ToM abilities in autistic children within a narrow age range of 4–6 years. In this study, neurotypical children (n = 39) and verbal autistic children (n = 61) were compared on a battery of tasks assessing various aspects of ToM (O’Brien et al., 2011; Wellman & Liu., 2004). They found that having a sibling, number of siblings, and having younger and older siblings were positively associated with ToM in a sub-group of autistic children without sibling recurrence but not in the entire sample of autistic children. In other words, for autistic children with non-autistic sibling/s (but not for those with autistic siblings), the presence of siblings was associated with better ToM performance.

In sum, this literature supports, albeit indirectly, a hypothesis of a positive effect of non-autistic siblings on cognitive empathy in autistic children. However, it is unclear whether this effect of siblings on cognitive empathy in autistic children also extends into adulthood, as has been found for typical individuals (Lo & Mar, 2022). In addition, to the best of our knowledge, no study has examined whether the positive effect of siblings on cognitive empathy in autistic children extends to the multidimensional nature of empathy beyond the cognitive aspect, as measured in ToM literature.

In the present study, we aimed to investigate the effect of siblings on empathy in autistic children and adults, using a measure designed to capture both the cognitive and affective components of empathy: the Empathy Quotient (EQ; Baron-Cohen & Wheelwright, 2004). The EQ provides a reliable and valid way to measure global empathy in typical and clinical populations in both the adults’ self-report version (Allison et al., 2011; Lawrence et al., 2004), and the children’s caregiver report version (EQ-C; Auyeung et al., 2009). We hypothesized that autistic people with siblings would score higher on empathy compared to those with no siblings, and specifically that:

1. Autistic children who have siblings will score higher on a caregiver report measure of empathy compared to autistic children who have no siblings.
2. Autistic adults who have siblings will score higher on both a self-report measure of empathy (EQ) and a performance measure of cognitive empathy (RMET) compared with autistic adults with no siblings.

Considering the previous literature on sex differences in empathy, indicating higher empathy in females on average compared to males (Baron-Cohen & Wheelwright, 2004), we further tested if there was an effect of sex or interaction between sex and group (having siblings vs. not having siblings) to test if there are different effects of having a sibling on empathy for autistic males and females.

## Methods

### Participants

The study included N = 1862 autistic individuals. Data were collected from the Cambridge Autism Research Database (CARD) (www.autismresearchcentre.com), with ethical approval from the University of Cambridge Psychology Research Ethics Committee (Pre.2013.06). Data for the present study were retrieved from CARD in January 2020.

Parents reported on their autistic children’s diagnosis, and autistic adults self-reported their diagnosis, age, and sex at birth. Additional demographic data collected from participants included the number of siblings in the family and whether any of the siblings were diagnosed with autism. As the present study focused on empathy and siblings, the CARD database was searched to identify those who had data for the parent-reported versions of the Empathy Quotient (EQ; Auyeung et al., 2009, 2012) and to identify autistic adults with self-report EQ data (Baron-Cohen & Wheelwright, 2004), and data from the Reading the Mind in the Eyes Test (RMET). Individuals with missing or inconsistent demographic data about the presence of siblings were excluded, as were those with autistic siblings. This resulted in two cohorts of:

1. Autistic children (*N* = 939; 16.40% females; age range 4–11 years old, *M*_*age*_ = 7.35 years, *SD* = 2.15; 21.73% of whom had no siblings).
2. Autistic adults (*N* = 736; 52.58% females; age range 18–81 years old, *M*_*age*_ = 37.05 years, *SD* = 12.39; 15.08% of whom had no siblings).

Participants in both cohorts were unique participants, i.e., they each only provided one data point for the study.

## Measures

### Empathy Quotient (EQ)

For adults, empathy was measured using the self-report version of the EQ (Baron-Cohen & Wheelwright, 2004). This questionnaire contains 40 items designed to measure empathy on a 4-point scale. On each item, a person can score 2 (if the respondent strongly agrees with the statement), 1 (if the respondent slightly agrees), or 0 (if the respondent does not agree). The range of scores on the EQ is 0 to 80. The EQ demonstrated high internal reliability (Cronbach’s alpha = 0.92) and high test–retest reliability (r = 0.97) (Baron-Cohen & Wheelwright, 2004; Lawrence et al., 2004). For children, empathy was measured using a parent-report version of the EQ that was adapted from the adult EQ by rephrasing questions to an age-appropriate level but kept as close to the adult versions as possible, with most questions aimed at the same behaviours (EQ-Child [EQ-C]: Auyeung et al., 2009). The EQ-C comprises 27 items, and the maximum score on the EQ-C is 54. Auyeung et al. (2009) reported high internal consistency (Cronbach’s alpha = 0.93), and good test–retest reliability (r = 0.86) for the EQ-C. A recent systematic review of measures of empathy in children and adolescents (Sesso et al., 2021) concluded that the EQ-C is a useful instrument that has been validated in autistic and non-autistic children and adolescents, demonstrating the highest internal consistency among the reviewed measures and a good test–retest index. The EQ and the EQ-C show clear sex differences (on average female advantage), and autistic people score lower than non-autistic people (Auyeung et al., 2009; Baron-Cohen et al. 2003; Baron-Cohen & Wheelwright 2004; Carroll & Chiew 2006; Lai et al. 2011; Wheelwright et al. 2006).

### Reading the Mind in the Eyes Test (RMET)

This 36-item task requires participants to infer mental states solely from photos of a person’s eyes. Participants are presented with a photograph of the eyes region of the face and must choose one of four adjectives or phrases (forced-choice words) to describe the mental state of the person pictured. For each item, only one response option is correct. Mental state words for response options were generated by the developers of the task and were then piloted on a group of eight judges until between ‘judges’ agreement was reached for each item on both the correct response option (by at least 5 out of the eight judges) and foils (no more than two judges picked any single foil). In the next step, the final items were established based on consensus from a large population study. The RMET score is the sum of correct answers, ranging from 0 to 36 (Baron-Cohen et al., 2001; Warrier et al., 2017). The RMET shows, on average clear sex differences (female advantage) (Greenberg et al., 2023; Warrier et al., 2017), and autistic people score lower than non-autistic people (Baron-Cohen et al., 2015). The RMET has been evaluated in hundreds of studies and has been found to have good reliability (Fernández-Abascal et al., 2013; Greenberg et al., 2023; Lombardo et al., 2007; Vellante et al., 2013). Validation of the psychometric properties of the RMET was evident in large-scale studies (Greenberg et al., 2023), and the National Institute of Mental Health (NIMH) Research Domain Criteria (RDoC) lists the RMET as one of the two recommended tests for the measurement of individual differences in “understanding mental states” (https://www.nimh.nih.gov/about/advisory-boards-and-groups/namhc/reports/behavioral-assessment-methods-for-rdoc-constructs). For this study, only data for adults on the RMET were analyzed.

### Data Analysis

The two cohorts (children; adults) were divided into two groups: those with siblings (Sib group) and those without siblings (No-sib group). In the autistic children cohort, the No-sib group consists of 204 participants (17.16% females), and the Sib group consists of 735 participants (16.19% females). In the adult cohort, the No-sib group consists of 111 participants (56.8% females), and the Sib group consists of 625 participants (51.8% females). Statistical tests that are appropriate for unequal sample sizes were used to compare group means on each measure in both cohorts. The Welch t-test was used to directly compare group means, and a 2-way ANOVA was utilized to examine the effects of group (with or without siblings), reported sex (male, female), and any interaction (Langsrud, 2003). Statistical analyses were conducted using RStudio based on R software (R Core Team, 2019).

## Results

Descriptive statistics are summarized in Table 1.

**Table 1:** Descriptive statistics of the children and the adults cohorts

		Children *N* = 939; ages 4–11		Adults *N* = 736; ages 18–72		
		*n*	EQ *M*(*SD*)	*n*	EQ *M*(*SD*)	RMET *M*(*SD*)
All		939	14.48 (7.26)	736	19.61 (12.40)	23.31 (6.76)
Sib	All	735	13.91 (6.84)	625	19.44 (12.42)	23.33 (6.70)
	Females	119	15.17 (6.87)	324	22.17 (14.10)	23.45 (6.97)
	Males	616	13.66 (6.81)	301	16.51 (9.55)	23.21 (6.40)
No-sib	All	204	16.57 (8.31)	111	20.57(12.35)	23.16 (7.11)
	Females	35	17.09 (8.56)	63	22.65 (13.20)	24.45 (6.41)
	Males	169	16.46 (8.28)	48	17.8 (10.70)	21.4 (7.65)

### Children

**Fig. 1:**
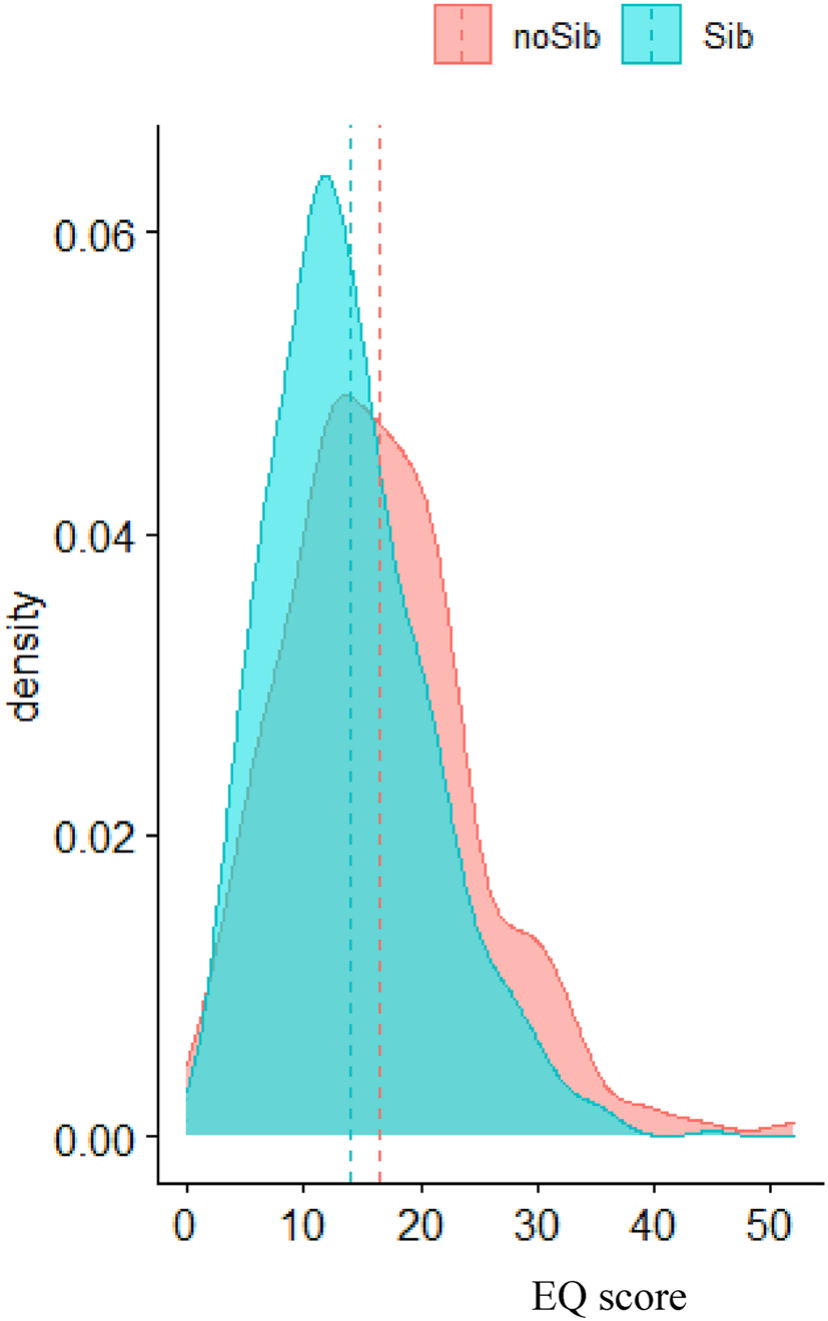
Density plot of EQ scores by group for the autistic children cohort. The x-axis shows the EQ score value, and the y-axis shows the relative frequency. The turquoise curve represents the group of autistic children with siblings (Sib), and the red curve represents the group of autistic children without siblings (No-sib). The dashed lines represent the groups’ means

The Welch t-test testing group differences (Sib or No-Sib) of EQ scores found a statistically significant group difference (difference = 2.66, 95% CI [1.41, 3.91], *t*_(283.70)_ = 4.20, p < 0.001; *Cohen’s d* = 0.50, 95% CI [0.26, 0.73]). Figure 1 shows density curves and means differences between the two groups.

Results of the ANOVA indicated a main effect of group (No-sib vs. Sib) (*F*_(1,935)_ = 22.00, *p* < 0.001; *η*^2^ = 0.02, 95% CI [0.00, 1.00]), a main effect of sex (*F*_(1,935)_ = 4.27, *p* = 0.04; *η*^2^ = 0.00, 95% CI [0.00, 1.00]) but no interaction between group and sex (*F*_(1,935)_ = 0.34, *p* = 0.56). Thus, contrary to the hypothesis, the results indicated that autistic children with no siblings scored higher on average on the EQ, compared to autistic children with siblings.

This effect also held when dividing the cohort into sub-groups according to birth order, with only children scoring higher on the EQ than those who had either younger, older, or younger and older siblings (*F*_(4,934)_ = 5.67, *p* < 0.001; *η*^2^ = 0.02, 95% CI [0.00, 1.00]. The difference in mean EQ scores between only children (*M* = 16.57) and the small group of children with co-twins (*n* = 20; *M* = 14.30) was not significant within the multiple comparisons. No other differences between groups by birth order were found. Tables 2 and 3 show descriptive statistics for EQ scores as a function of birth order and Tukey HSD test for multiple comparisons results).

**Table 2:** EQ Mean scores & SD for Children as a function of reported birth order

Group (by birth order)	*n*	*M*_*EQ score*_	*SD*
Only children	204	16.57	8.31
Have older sibling/s	254	13.59	6.54
Have younger sibling/s	346	14.07	6.96
Have younger and older siblings	115	14.05	7.24
Twins	20	14.30	6.24

**Table 3:** Tukey HSD test for multiple comparisons results for EQ scores for children by birth order

Groups (by birth order) comparisons	Difference	95% CI		p adjusted
		Lower	Upper	
Only children vs. have older sibling/s	2.98201328	1.13487310	4.829153	0.0001113
Only children vs. have younger sibling/s	2.49637312	0.76206090	4.230685	0.0008515
Only children vs. have younger and older siblings	2.51645354	0.22542550	4.807482	0.0230244
Have younger sibling/s vs. have older sibling/s	0.48564016	− 1.1377383	2.109019	0.9252424
Have younger and older siblings vs. have older sibling/s	0.46559740	− 1.7426813	2.673801	0.9785356
Have younger sibling/s vs. have younger and older siblings	0.02008040	− 2.0946867	2.134848	0.9999999
Twins vs. have older sibling/s	0.71338583	− 3.8495303	5.276302	0.9930472
Twins vs. younger and older siblings	0.24782609	− 4.5121200	5.007772	0.9999078
Twins vs. have younger sibling/s	0.22774566	− 4.2906739	4.746165	0.9999190
Twins vs. only children	− 2.26862745	− 6.8721788	2.334924	0.6618496

We also had a small (not adequately powered) additional cohort of autistic adolescents (*N* = 232; 48 of whom had no siblings; 29.31% females; age range 12–15 years old) on which we conducted further exploratory analyses to explore if this effect replicates in older children. EQ was similarly measured by parental report (EQ-Adolescent: Auyeung et al., 2012). Results from this exploratory analysis implied a similar trend to results found in the children cohort. The analyses and the results are detailed in the supplementary materials.

### Adults

#### Empathy Quotient (EQ)

**Fig. 2:**
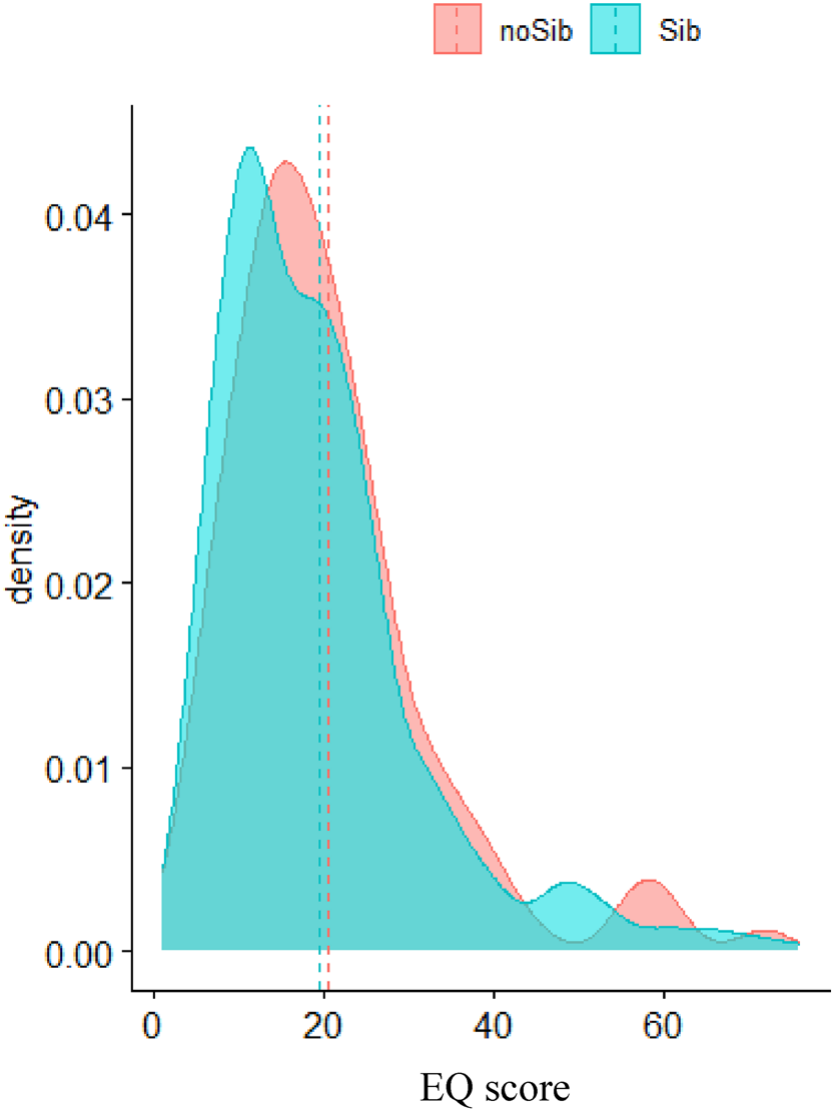
Density plot of EQ scores by group for the adults cohort. The x-axis shows the EQ score value, and the y-axis shows the relative frequency. The turquoise curve represents the group of autistic children with siblings (Sib), and the red curve represents the group of autistic children without siblings (No-sib). The dashed lines represent the groups’ means

The Welch t-test testing group differences (Sib or No-Sib) of EQ scores found no main effect of group (difference = 1.12, 95% CI [− 1.39, 3.64], *t*_(152.21)_ = 0.88, *p* = 0.378). Figure 2 shows density curves and means differences between the two groups.

Results of the ANOVA found no main effect of group (Sib vs. No-sib) (*F*_(1,732)_ = 0.81, *p* = 0.368), and a small but significant main effect of sex (*F*_(1,732)_ = 38.30, *p* < 0.001; *η*^2^ = 0.05, 95% CI [0.03, 1.00]), with no interaction between group and sex (*F*_(1,732)_ = 0.11, *p* = 0.11).

#### Reading the Mind in the Eyes Test (RMET)

**Fig. 3:**
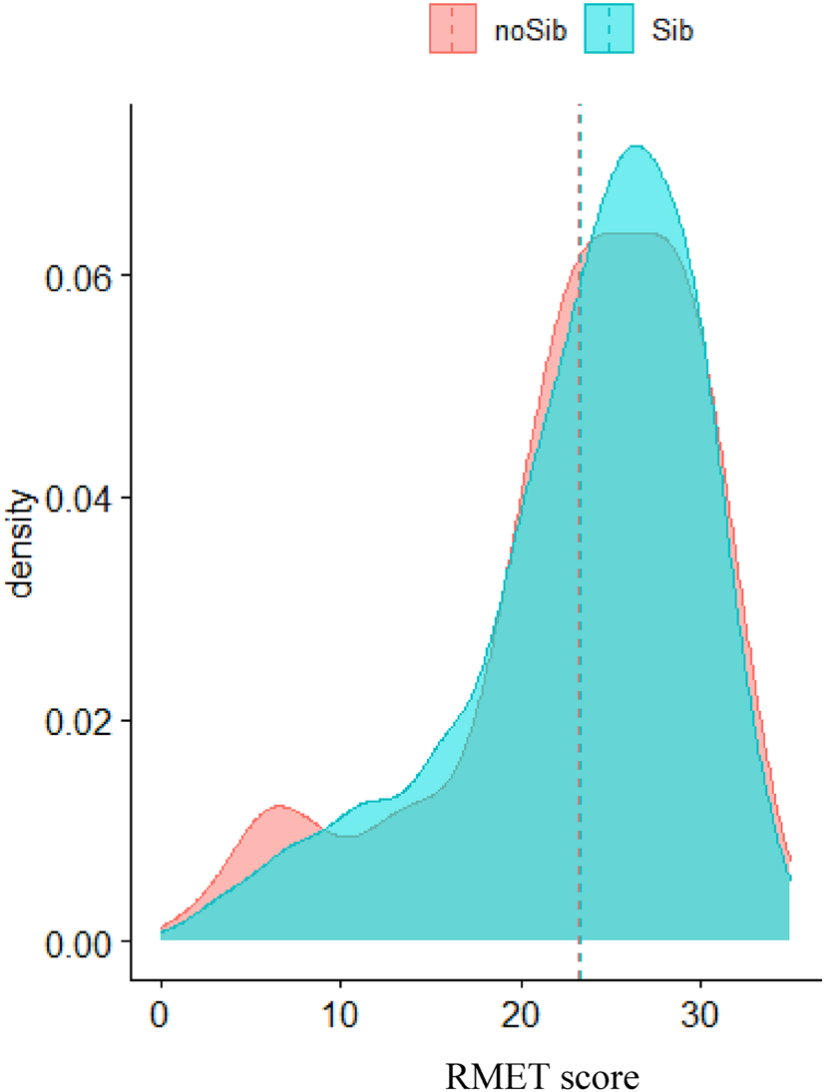
Density plot of RMET scores by group for the adults cohort. The x-axis shows the RMET score value, and the y-axis shows the relative frequency. The blue curve represents the group of autistic adults with siblings (Sibs), and the red curve represents the group of autistic adults without siblings (No-sibs). The dashed lines represent the groups’ means

The Welch t-test testing group differences (Sib or No-Sib) of RMET scores found no effect of group (difference = − 0.17, 95% CI [-1.61, 1.26], *t*_(146.79)_ = − 0.24, *p* = 0.813). Figure 3 shows density curves and means for the two groups.

Results of the ANOVA found no main effect of group ((*F*_(1,732)_ = 0.06, *p* = 0.804), or sex (*F*_(1,732)_ = 1.77, *p* = 0.184), and with a very small interaction effect between group and sex (*F*_(1,732)_ = 4.10, *p* = 0.043; *η*^2^ < 0.001,^1^ 95% CI [0.00, 1.00]).

### Further Analysis by Sex

**Fig. 4:**
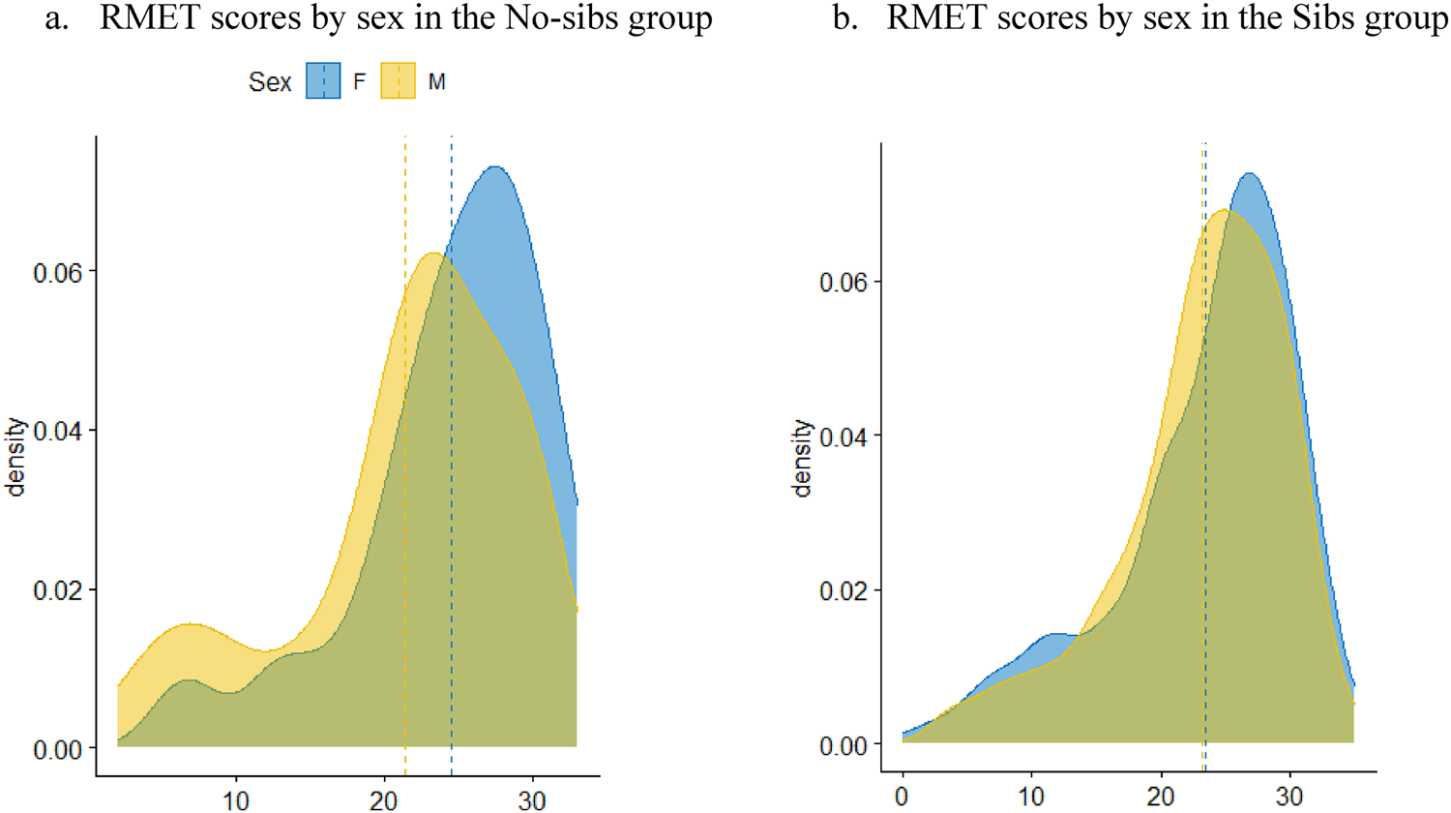
Density plot of RMET scores by sex for only the group of participants without siblings (a. No-sibs) and for only the group of participants with siblings (b. Sibs) in the adults’ cohort. The x-axis shows the RMET score value, and the y-axis shows the relative frequency. The blue curve represents autistic females, and the yellow curve represents autistic males with no siblings. The dashed lines represent the groups’ means

**Fig. 5:**
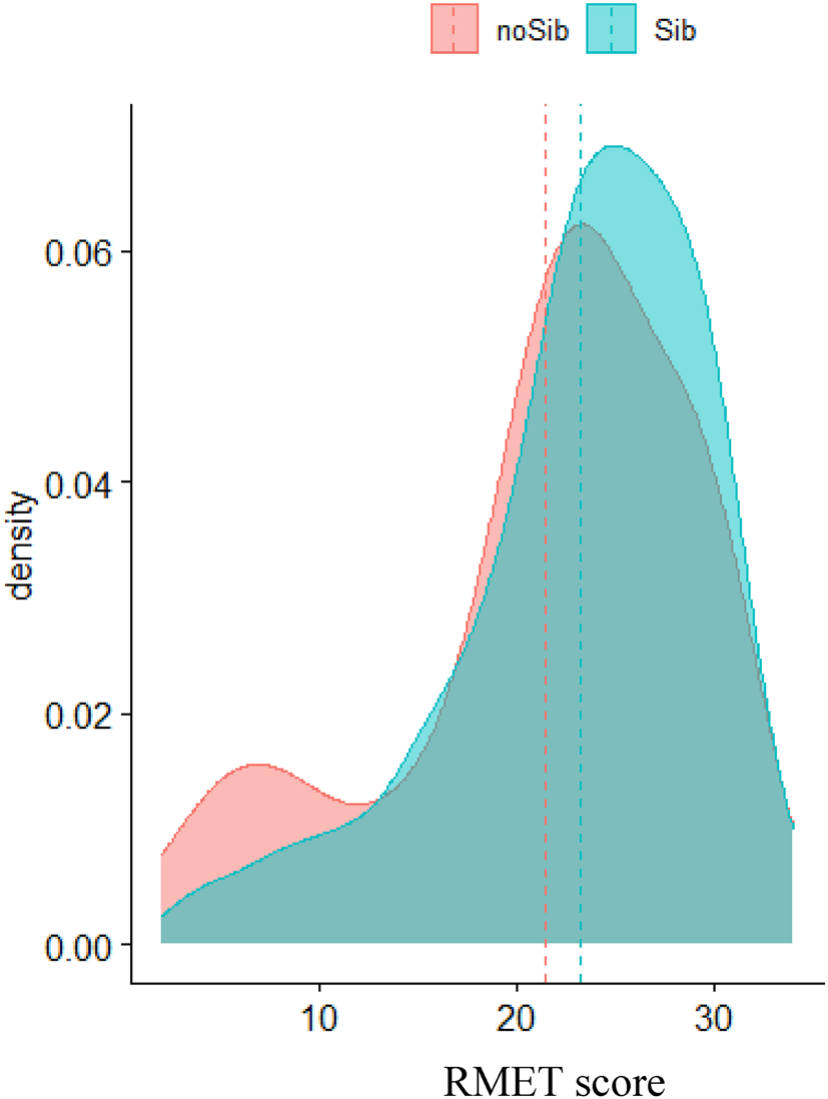
Density plot of RMET scores by group for only the males in the adults’ cohort. The x-axis shows the RMET score value, and the y-axis shows the relative frequency. The turquoise curve represents the group of male autistic adults with siblings (Sib), and the red curve represents the group of male autistic adults without siblings (No-sib). The dashed lines represent the groups’ means

Figure 4 shows the density plot of RMET scores by sex for the autistic participants without siblings and those with siblings. The Welch Two Sample t-test testing sex differences of RMET score in the No-sib group suggests that in this group, females scored higher than males (difference = 3.08, 95% CI [0.36, 5.79], *t*_(90.94)_ = 2.25, *p* = 0.027; *Cohen’s d* = 0.47, 95% CI [0.05, 0.89]). In contrast, there was no sex difference between males and females on the RMET for participants with siblings (difference = 0.24, 95% CI [− 0.81, 1.29], *t*_(622.91)_ = 0.45, *p* = 0.652; *Cohen’s d* = 0.04, 95% CI [− 0.12, 0.19]). When comparing males with and without siblings (see Fig. 5.) there was no significant difference (difference = 1.79, 95% CI [0.54, 4.12], *t*_(57.95)_ = 1.54, *p* = 0.129; *Cohen’s d* = 0.40, 95% CI [0.12, 0.92]).

## Discussion

This study aimed to test the effect of having siblings on empathy in autistic people. We predicted that autistic individuals with siblings would score higher on measures of empathy than those without siblings. However, this hypothesis was not supported by our data from two cohorts of autistic children (based on parent-reported empathy) and autistic adults (based on self-report empathy and a performance task designed to measure cognitive empathy). The data also suggested that sex plays a role in the effect of siblings on empathy in autistic individuals, as indicated by an interaction effect with a very small effect size. While autistic males scored lower than autistic females on both the EQ and the RMET, autistic males with siblings did not score lower than females on the cognitive empathy performance test (RMET). This finding that implies a positive effect of siblings on cognitive empathy in autistic males requires further investigation.

Contrary to the hypothesis of this study and in sharp contrast to the literature indicating a positive effect of siblings on social-cognitive outcomes in autistic children (Ben-Itzchak et al., 2016; Ben-Itzchak et al., 2019; Matthews et al., 2013; Matthews & Goldberg, 2018; Rosen et al., 2022), in the present sample, autistic children who grew up with siblings were reported by their parents to be *less* empathic compared to those without siblings. It could be argued that this effect of having siblings on empathy in autistic individuals is an indication of support for the theory of resource dilution. For example, parents who need to divide their attention or other resources across multiple children in the family do not fully allocate them to support the development of the autistic child. This explanation was previously suggested by O’Brien et al. (2011), who found a disadvantage of having an older sibling and a slight advantage for having a younger sibling on ToM performance in autistic children (albeit based on relatively small sample sizes of 15 only-children, 13 children with older siblings, and 22 children with younger siblings). O’Brien et al. (2011) pointed out that it is likely that a first-born autistic child (with only younger sibling/s) is less impacted by such resource dilution in the first years of life, before the birth of another child in the family, and thus, is not impacted by the negative effect of siblings as a non-first born child (with only older sibling/s). However, the resource dilution theory is not a sufficient explanation for the findings of the present study. We found that autistic children with either older sibling/s, younger sibling/s, or younger and older siblings were all reported by their parents to have lower empathy scores than autistic children with no siblings. Furthermore, there was no indication of this effect in autistic adults on the EQ and the RMET.

Another explanation for the greater reported empathy in autistic children without siblings might be related to the nature of the parent report measure of empathy. Parents may report their autistic children’s empathic abilities relative to their other children. Those parents who do not have another child have nothing against which to compare. A bias in parent reports of children was termed a contrast effect, that is, a tendency to exaggerate differences between siblings. A contrast effect has primarily been reported in twin studies, especially those exploring genetic and environmental contributions to variation in child temperament or psychopathology when using parental questionnaires (Eaves et al., 2000; Simonoff et al., 1998). This was also later reported for non-twin siblings, regarding children’s temperament (Saudino et al., 2004; Saudino, 2003a, 2003b).

The possibility of contrast effects in parent reports might be more than a psychometric limitation, as contrast effects may represent a meaningful construct in guiding parents’ behaviour towards their children. Put another way, parents’ perceptions of their children might impact social interactions in the family. In the present study, it is possible that a contrast effect encourages parents to underestimate their autistic child’s empathy.

Rosen et al. (2022) found a positive effect of siblings on the outcomes of autistic children’s adaptive functioning using a semi-structured interview (VABS; Sparrow et al.,  1984, 2005). This study used data from the VABS longitudinally to assess growth in daily living skills. This means that the child’s progress was determined by changes on the VABS for each child across more than one time point, and the child’s performance at each point is compared to their own previous performance and not to another child, thus making a contrast effect less likely to interfere with the results. Unlike the Rosen et al. (2022) study, the present study required parents to report on their children’s empathy, thus requiring them to speculate on their children’s mental states. It could be that parents have more limited access to their autistic children’s mental states compared to the non-autistic children in the family, perhaps due to social communication difficulties between the autistic child and the parent. Having social communication challenges with one child in the family compared to the other/s could become a fertile context for a contrast effect to develop when reporting the autistic child’s empathy.

Importantly, there is evidence for greater cognitive empathy in siblings of children with disabilities (Rum et al., 2022) and specifically in siblings of autistic children (Shivers et al., 2019). It might be that a close relationship with someone with a ‘different’ mind provides opportunities to practice empathy, or it could be that the typical sibling develops greater empathy, facilitating communication with their brothers or sisters, and overcoming challenges in their relationships with their autistic siblings. It is also possible that siblings of autistic children have a greater caring role, which nurtures empathy (Cuskelly & Gunn, 2003). Further, being exposed to a sibling’s disabilities and challenges may enhance a general empathic sensitivity. Even though the mechanism is not yet clear, this novel line of research converges with previous qualitative findings and anecdotal self and parental reports on siblings of children with disabilities as human beings with enhanced empathic abilities (e.g., Flaton, 2006; Taunt & Hastings, 2002). It could be that when parents report on the empathy of their autistic children and they have another child demonstrating enhanced empathy, the contrast effect between the children is amplified. In future studies, it would be interesting to investigate empathy in autistic children with and without siblings using self-reports, observations, and other behavioural measures or to consider the reports of another informant, such as, for example, teachers. It is also important to explore parents’ perceptions of their autistic children’s abilities in various familial constellations.

Interestingly, an interaction effect between sex and group (with/without siblings) was found for the performance of autistic adults on the RMET. Males scored lower than females in the no-siblings group, while males with siblings did not score lower than females with siblings. The very small effect size of this interaction effect implies that caution is needed in interpreting these results and their implications in “real life”. However, further analysis by sex may shed some more light on the trends indicated by the results and highlight important future research directions. Overall, and consistent with previous literature (e.g., Baron-Cohen & Wheelwright, 2004), males scored lower than females on all measures of empathy in the present study regardless of sibling status, with the exception of adult males who grew up with siblings that did not score lower than females in the RMET. This result echoes the findings from a recent study of *n* = 1,792 typical adults indicating that typical males, on average, have better mentalizing abilities if they have siblings, but this advantage is attenuated for typical females (Lo & Mar, 2022). It is possible that males benefit more than females from growing up with siblings. It could also be that females benefit more from learning from others in the social environment beyond their nuclear family, whereas for males, the role of siblings as socialization agents and social partners is more vital. However, a direct comparison between males who grow up with and without siblings in the present sample did not reach statistical significance. This could be due to a lack of power to detect a statistically significant difference due to the relatively small number of autistic males without siblings in the present sample. In addition, we did not have data regarding the sex of the siblings in this cohort. The role of sex in the association between siblings and empathy of autistic individuals could lie not only in the sex of the autistic individual but also in the sex of the sibling or even in both siblings’ sex match, i.e., the pair being same-sex or an opposite-sex siblings pair (see, for example, Wright-Cassidy et al., 2005). In light of these limitations, further replication with larger numbers and data on both siblings’ sex is clearly warranted to better understand the role of sex in the effect of siblings on empathy in autistic people and specifically to directly examine the hypothesis of a positive effect of siblings on empathy in autistic male individuals.

Other limitations of this study include that this was a secondary analysis of previously collected data, and we did not have available data on the participants’ language and social communication skills. We were, thus, limited in examining the role of these variables as mediators or moderators in the effect of siblings on empathy in our sample. These questions should be addressed in future research. Importantly, information about siblings, such as siblings’ age and age gaps between the autistic participants and their siblings, and whether they experienced childhood together (see McAlister & Peterson, 2006, 2007), was also lacking, as well as the quality of the sibling relationship. Individuals with autistic siblings were excluded from the study, but it remains possible that the siblings may have had other conditions, including high autistic traits. Further information about sibling characteristics should be acquired in future studies on this topic. Conversely, relying on secondary data that had not been collected to target the effect of siblings on empathy in autistic individuals may have been less prone to bias. For example, the measures were collected without prompting participants that the study was focusing on the presence or absence of siblings, thus reducing the likelihood of exacerbating a contrast effect between autistic individuals and their siblings.

In future replications, using other measures of empathy will also be valuable. For example, Muncer and Ling (2006) noted that the EQ is a valid self-report measure of general trait empathy, but to capture empathic abilities, i.e., the ability to perform tasks that require using the multifacet concept of empathy—an ability-based measure might be needed. One such measure is empathic accuracy, operationalized in various ecological paradigms (for review, see Rum & Perry, 2020).

It is also important to note that the adults’ cohort in the present study was composed of participants who were able to participate online. That means they could read, give consent, and complete a self-report questionnaire and a behavioral task. The results and their interpretations and generalizability must, thus, be considered according to the sample characteristics. It is important to further examine the effect of siblings on empathy and other social cognitive outcomes in autistic adults who cannot independently complete such participation.

Despite these limitations, and although the initial research hypothesis was not supported, this study contributes to a deeper understanding of siblingship and empathy in the context of autism. Our findings suggest the possibility of a contrast effect in parents’ perceptions of their autistic children when there are other brothers or sisters in the family. Considering previous literature, arguably, this contrast effect points towards parents underestimating their autistic children’s empathy when non-autistic siblings are present in the family. This possible bias in parental perception should be considered in research as well as in clinical and educational work with autistic individuals and their families. It will be interesting to explore the possibility of a contrast effect in parental reports for autistic adults. Our results also imply that, similarly to findings from typical population, having siblings might positively affect autistic male adults’ empathy, but autistic female adults with siblings might not exhibit such an advantage. Beyond the theoretical contribution to understanding the role of sex in empathy and siblinghood in the context of autism, this finding also shows that empathy is shaped by various aspects of siblinghood in autistic individuals in similar ways as it does for non-autistic individuals. An interesting future direction will be exploring associations between empathy and aspects of the sibling relationship, such as warmth, closeness, and conflict. Future work should also incorporate a wide range of empathy measures and informants for autistic children and adults and directly examine parents’ perspectives on their sons’ and daughters’ empathic abilities.

In closing, this study implies that growing up with a sibling, as opposed to being an only child, might matter for empathy in autistic individuals (depending on their gender), and it might also matter for how their parents perceive their empathic abilities.

## Footnotes

1 Note that the value of *η*^2^ was very close to 0: 8.69e-05, meaning that despite the statistical significance caution is needed in interpreting the meaning and implication of the effect.
